# Precision Medicine in Alzheimer’s Disease: Investigating Comorbid Common Biological Substrates in the Rat Model of Amyloid Beta-Induced Toxicity

**DOI:** 10.3389/fphar.2021.799561

**Published:** 2022-01-03

**Authors:** Maria Grazia Morgese, Maria Bove, Lorenzo Di Cesare Mannelli, Stefania Schiavone, Anna Laura Colia, Stefania Dimonte, Emanuela Mhillaj, Vladyslav Sikora, Paolo Tucci, Carla Ghelardini, Luigia Trabace

**Affiliations:** ^1^ Department of Clinical and Experimental Medicine, University of Foggia, Foggia, Italy; ^2^ Pharmacology and Toxicology Section, Department of Neuroscience, Psychology, Drug Research and Child Health (NEUROFARBA), University of Firenze, Firenze, Italy; ^3^ Department of Pathology, Sumy State University, Sumy, Ukraine

**Keywords:** pain, amyloid beta, depression, serotonin, kynurenine, melatonin, glutamate, precision medicine

## Abstract

Alzheimer’s disease (AD), one of the most widespread neurodegenerative disorder, is a fatal global burden for the elder population. Although many efforts have been made, the search of a curative therapy is still ongoing. Individuating phenotypic traits that might help in investigating treatment response is of growing interest in AD research. AD is a complex pathology characterized by many comorbidities, such as depression and increased susceptibility to pain perception, leading to postulate that these conditions may rely on common biological substrates yet to be determined. In order to investigate those biological determinants to be associable with phenotypic traits, we used the rat model of amyloid beta-induced toxicity. This established model of early phase of AD is obtained by the intracerebroventricular injection of soluble amyloid beta1-42 (Aβ) peptide 7 days before performing experiments. In this model, we have previously reported increased immobility in the forced swimming test, reduced cortical serotonin levels and subtle alterations in the cognitive domain a depressive-like phenotype associated with subtle alteration in memory processes. In light of evaluating pain perception in this animal model, we performed two different behavioral tests commonly used, such as the paw pressure test and the cold plate test, to analyze mechanical hyperalgesia and thermal allodynia, respectively. Behavioural outcomes confirmed the memory impairment in the social recognition test and, compared to sham, Aβ-injected rats showed an increased selective susceptibility to mechanical but not to thermal stimulus. Behavioural data were then corroborated by neurochemical and biochemical biomarker analyses either at central or peripheral level. Data showed that the peptide injection evoked a significant increase in hypothalamic glutamate, kynurenine and dopamine content, while serotonin levels were reduced. Plasma Cystatin-C, a cysteine protease, was increased while serotonin and melatonin levels were decreased in Aβ-injected rats. Urinary levels paralleled plasma quantifications, indicating that Aβ-induced deficits in pain perception, mood and cognitive domain may also depend on these biomarkers. In conclusion, in the present study, we demonstrated that this animal model can mimic several comorbid conditions typical of the early phase of AD. Therefore, in the perspective of generating novel therapeutic strategies relevant to precision medicine in AD, this animal model and the biomarkers evaluated herein may represent an advantageous approach.

## Introduction

Alzheimer’s disease (AD) is the most common form of dementia in the elderly. It is nowadays becoming more evident that AD is heterogeneous in many aspects, ranging from biomolecular or clinical manifestations, thus indicating that AD cannot be explained with a single pathological process (Ferrari and Sorbi, 2021). In this light, studying differences in disease symptomatology, treatment responses may represent an innovative field of study for supporting and implementing precision medicine in AD. Indeed, this complex neurological disorder is often accompanied by a variety of comorbidities. Among these, depression is highly frequent in the way that some Authors have hypothesized that depressive symptoms may represent an early manifestation of this neurodegenerative disease (Pomara and Sidtis, 2010). Interestingly, the prevalence of chronic pain in dementia is quite elevated and it is esteemed to be between 30 and 80% (Defrin et al., 2015). On the other hand, several lines of evidence have reported alterations of pain perception in different psychiatric disorders (Lautenbacher and Krieg, 1994; Malejko et al., 2020). In particular, the pain experience has been frequently associated with emotional and cognitive dysfunctions, as well as to depressive states (Moriarty et al., 2011; Bushnell et al., 2013). Indeed, the comorbidity of pain and depression has been described as the “pain-depression syndrome or pain-depression dyad” (Thompson et al., 2016). More specifically, depression may induce a reduction of the pain threshold (Torta and Munari, 2010). This suggests a possible overlapping of neurobiological and molecular mechanisms underlying this comorbidity (Li, 2015), whose understanding may be crucially provided by preclinical investigations using animal models of depressive-like state. In this regard, we have previously reported that rats receiving an intracerebroventricular (icv) injection of soluble amyloid beta1-42 (Aβ) peptide showed increased immobility frequency in the forced swimming test, mimicking a behavioral despair state, also accompanied by neurochemical alterations, as well as changes in neurotrophin levels (Colaianna et al., 2010; Schiavone et al., 2017). Furthermore, in this animal model, depressive like symptoms are accompanied by subtle changes in spatial memory, mimicking the early manifestation of AD onset, featured by higher Aβ levels and alterations in emotional and cognitive domains. In this regard, we have previously fully validated this model. Immunohistochemical analyses were also previously carried out after the peptide injection (Trabace et al., 2007), and the peptide solution was fully evaluated by atomic force and transmission electron microscopy (Morgese et al., 2015). In particular, systemic soluble Aβ levels were still highier in treated injected rats 7 days after central administration of the peptide, while immunohistochemical analyses reveiled no gross signs of neurodegeneration within the area of Aβ diffusion in the periventricular parenchyma (Trabace et al., 2007; Morgese et al., 2015; Schiavone et al., 2017; Morgese et al., 2018). Therefore, in this model we generally assume that the supraphysiological levels of the peptide lead to profound unbalance in several brain circuitries (see (Trabace et al., 2007; Preda et al., 2008; Mura et al., 2010) for reference). Thus, from a behavioral and neurobiological point of view, this model can be considered as a valid animal model of early AD phase.

A key brain region involved in pain perception, particularly in its descending modulation, is the hypothalamus (Puopolo, 2019), which has also been reported to play key functions in the development of different depressive symptoms, acting as a crucial hub in a network of neurocircuits modulating these two comorbidities (Bao and Swaab, 2019). Interestingly, alteration in the hypothalamic area has also been reported in the early AD phase (Vercruysse et al., 2018). Of note, neurotransmitter systems implicated in pain perception, transmission and control physiologically overlap with those underlying the development of depressive disorders and cognition (Yang and Chang, 2019). Among them, dopaminergic pathways have been implicated in the central regulation and modulation of pain (Li C. et al., 2019). Conversely, nociceptive stimuli have been described to disrupt dopamine homeostasis in the central nervous system (Wood, 2008; Kim et al., 2015). Moreover, it has been reported that soluble Aβ could determine alterations in dopamine content in the rodent brain (Wu et al., 2007; Morgese and Trabace, 2019). Together with its crucial role in pain perception (Paredes et al., 2019), decreased central levels of serotonin (5-HT) have been widely described in depressive-like states induced by soluble Aβ (Colaianna et al., 2010; Schiavone et al., 2017; Morgese et al., 2018). In addition, drugs able to increase 5-HT levels can improve cognitive function in AD patients (Xie et al., 2019) and in rodent AD models (Ma et al., 2017). Increased levels of glutamate (GLU), a key player in the perception of pain and pain transmission from peripheral to central districts (Pereira and Goudet, 2018), were found in rodent brain following icv injection of soluble Aβ able to cause spatial memory impairment (Tucci et al., 2014), and whose accumulation might also lead to synaptic failure through disruption of the glutamatergic pathways (Findley et al., 2019). Of note, ketamine, an antagonist of the glutamatergic NMDA receptor, is able to reduce pain perception as well as depressive symptoms (Dowben et al., 2013; Hayley and Litteljohn, 2013), and in this model we have demonstrated that ketamine is able to prevent depressive-like phenotype in Aβ-treated rats (Schiavone et al., 2017).

Together with neuropathological dysfunctions at central levels, depression and AD have been described to be also associated with peripheral alterations. In particular, decreased plasma 5-HT levels have been detected in patients with major depression (Paul-Savoie et al., 2011). The 5-HT content in plasma has also been considered as a predictor of antidepressant treatment outcomes (Blardi et al., 2002; Holck et al., 2019; Sun et al., 2020). In addition, it has been suggested that the peripheral serotonergic system is negatively implicated in AD (Kumar et al., 1995). Moreover, a negative association has been found between peripheral 5-HT amount and pain perception after different noxious stimuli (Ernberg et al., 2000; Farhanchi et al., 2018). On the other hand, melatonin (MEL), a circadian rhythm–regulated hormone, has been shown to be protective in neurodegeneration associated with AD (Savaskan et al., 2005). Indeed, anti-amyloidogenic as well as free radical–scavenging properties have been reported for MEL leading to hypothesize that this molecule might represent a therapeutic candidate to inhibit AD progression (Rosales-Corral et al., 2012; Lin et al., 2013) or a valid biomarker for defining disease progression or therapeutic success (Nous et al., 2021).

Depressive symptoms have also been associated with increased peripheral levels of Cystatin-C (Cys-C), a low-molecular-weight protein, synthesized at a constant rate in all nucleated cells (Li H. et al., 2019; Huang et al., 2021). Cys-C has been thought to be crucially implicated in the development of soluble Aβ neuronal dysfunctions, also relating to cognitive impairment (Zeng et al., 2019). Of note, central and peripheral enhanced amount of this protein has been described as a pain biomarker (Mannes et al., 2003). Hence, in our animal model of early phase AD, we investigated possible alterations of pain threshold as possible comorbidity in the early AD phase and in the depressive-like state.

In addition, involvement of noradrenergic, dopaminergic, serotonergic, including the tryptophan derivate kynurenine (KYN), glutamatergic and gamma-aminobutyric acid (GABA)-ergic systems was assessed in the hypothalamus, an area whose dysfunction has been considered a putative driver of AD pathology (Ishii and Iadecola, 2015). Furthermore, possible alterations in peripheral, such as in plasma and in urine levels, of 5-HT, KYN, MEL and Cys-C amounts were also evaluated.

In this light, the establishment of an animal model that can allow to study different aspects of this neuropathology could represent a valid advantage in the perspective of building tailored treatment for AD patients, thus pursuing the goal of precision medicine for this complex neurologic and neuropsychiatric disorder.

## Materials and Methods

### Animals

Experiments were performed on a total number of 47 seven week-old male Wistar rats (Envigo, San Pietro al Natisone, Italy). Animals were constantly maintained under controlled conditions, with a room temperature of 22 ± 1°C, relative humidity of 55 ± 5% and a light/dark cycle of 12 h (light on from 7:00 a.m. to 7:00 p.m.). Rats were group-housed and food and water were provided *ad libitum*. We carried out all experimental procedures involving animals in conformity with the institutional guidelines of the Italian Ministry of Health (D.L. 26/2014), the Guide for the Care and Use of Mammals in Neuroscience and Behavioral Research (National Research Council 2004), the Directive 2010/63/EU of the European Parliament and of the Council of September 22, 2010, on the protection of animals used for scientific purposes, as well as the ARRIVE guidelines. The Italian Ministry of Health approved our experimental protocol (protocol number: B2EF8.15-aut.737-2017-PR). Animal welfare was daily monitored in order to detect any signs of animal suffering or distress during the entire experimental procedure. All efforts to reduce the number of animals used and their suffering were conducted.

### Aβ Administration

The human Aβ1–42 peptide (Tocris, Bristol, United Kingdom) was freshly prepared by using sterile double distilled water as vehicle (final concentration 4 μM), as previously described (Bove et al., 2018). An anesthetic cocktail solution (0.85 ml/kg) containing ketamine (100 mg/ml), xylazine (100 mg/ml) and acepromazine (10 mg/ml), dissolved in saline, was injected intraperitoneally in seven week-old rats. Rats were then secured in a stereotaxic frame (David Kopf Instruments, Tujunga, CA, United States) and underwent an icv infusion based on the following coordinates from bregma according to Paxinos and Watson rat brain atlas (Paxinos, 1986): AP = −0.5, ML = +1.2 and DV = −3.2. The incisor bar was set at −3.3 mm. A Hamilton micro-syringe was used to infuse 5 μl of soluble Aβ peptide, with an infusion rate of 2 μl/min over 2.5 min. Subsequently, the needle was left in place for 5 min in order to avoid elapsing. Vehicle was delivered to sham animals used as controls. Once dissecting the brain, it was verified whether the needle was placed in the correct way. Seven days after icv administration, experimental procedures were carried out.

### Behavioural Tests

#### Paw Pressure Test

The test was performed according to Di Cesare Mannelli et al. (Di Cesare Mannelli et al., 2015), by using an analgesimeter (Ugo Basile, Varese, Italy) to determine the nociceptive threshold in rats. This test is widely used as a test for hyperalgesia-like measurement (Deuis et al., 2017). Briefly, a blunt conical mechanical probe was used to apply an increasing pressure to the dorsal surface of the hind paw of slightly restrained rats. The constantly increasing pressure was applied until rats vocalized or showed a withdrawal reflex. Results were expressed in grams of tolerated mechanical pressure. The arbitrary cut-off value was set at 100 g and a blind observer collected the data.

#### Cold Plate Test

The test was performed by using a cold plate kept at a constant temperature (4 ± 1°C) as floor in a stainless steel box (12 cm × 20 cm × 10 cm), as reported by Pacini et al. (Pacini et al., 2016). This test is widely used as a test for allodynia-like measurement (Deuis et al., 2017). Each animal was placed in the box and the latency to first lick the hind paw, expressed in seconds, was recorded as a measure of pain-related behaviour. A cut-off time of 60 s was adopted.

#### Social Recognition Test

This test was used to analyze short-term memory by using a natural animal behaviour as social interacting with a similar (Mathiasen and Dicamillo, 2010) Briefly, rats were housed individually in plastic cages 3 days before the test. Juvenile rats used as stimulus animals were isolated in individual cages 30 min prior the beginning of the experiment. The social recognition test consisted in two trials of 5 min each, separated by an inter-trial of 30 min. During the first trial (T1) the stimulus animal is placed in the test animal home cage for 5 min. Then, an inter-trial of 30 min in which the stimulus returned to its cage was carried out. Subsequently, the trial 2 (T2) took place and the test animal was re-exposed to the stimulus animal. Time spent sniffing and social interacting, expressed in seconds, was scored by a blind observer. In order to exclude exploratory impairments, the time spent exploring the environment was also recorded.

### Post-mortem Analyses

At the end of the behavioural experiments, rats were euthanized, plasma was collected and brains were dissected by using a chilled rat brain matrix (World Precision Instruments, Inc. FL, United States) according to Paxinos and Watson rats brain atlas (Paxinos, 1986). The hypothalamus was weighed, frozen in liquid nitrogen and stored at − 80°C until *ex vivo* quantifications were performed.

#### High-Performance Liquid Chromatography (HPLC) Quantifications

Neurochemical analyses were performed by using HPLC. Hypothalamic samples were homogenized in 10 volumes (w/V) of 0.1 N perchloric acid, stored on ice for 30 min and then centrifuged at 10.000 × g for 10 min at 4°C, as previously described (Zotti et al., 2013). Plasma and urine samples were homogenized with 0.1 volumes (V/V) of 8% perchloric acid and centrifuged at 10.000 × g for 15 min at 4°C. Supernatants were then collected and HPLC analyses were carried out. Hypothalamic noradrenaline (NA), dopamine (DA) and 5-HT concentrations, plasma and urine 5-HT and 5-hydroxy-indole acetic acid (5-HIAA) levels were determined by using a LC18 reverse phase column (Kinetex, 150 mm × 4.2 mm, ODS 5 μm; Phenomenex, Castel Maggiore-Bologna, Italy). An electrochemical detector with a thin-layer amperometric cell (Ultimate 3000RS–ECD, Dionex, ThermoScientifics, Milan, Italy) at a working potential of 0.400 V was used. As previously described, KYN (Morgese et al., 2021b) and MEL were quantified with the same method by applying a working potential of 0.550 V. The flow rate was maintained at 0.7 ml/min by an isocratic pump (Shimadzu LC-10 AD, Kyoto, Japan). The mobile phase consisted of an aqueous solution of 75 mM NaH_2_PO_4_, 1.7 mM octane sulfonic acid, 0.3 mM EDTA, acetonitrile 10%, buffered at pH 3.0. Chemicals and reagents were purchased from Sigma Aldrich (Sigma Aldrich Milan Italy). Chromeleon software (version 6.80, Thermo Scientific Dionex, San Donato Milanese, Italy) was used for data analyses. The 5-HT turnover was calculated as 5-HIAA/5-HT ratio. Sample concentrations were expressed as fmol/mg of tissue. A fluorescence detector (emission length, 460 nm; excitation length, 340 nm, Jasco, Tokyo, Japan), after derivatization with *o*-phthalaldehyde/mercaptopropionic acid, was used to determine GLU, glutamine and GABA hypothalamic concentrations, as previously described (Bove et al., 2018). Chromatographic evaluation was accomplished by using an ODS-3 column (Kinetex 150 mm × 3 mm, ODS 5 μm; Phenomenex, Castel Maggiore-Bologna, Italy) with a gradient mobile phase (50 mM sodium acetate buffer, pH 6.95, with methanol increasing linearly from 2 to 30% (v/v) over 40 min. The flow rate was set at 0.5 ml/min. Data were acquired and integrated by using Borwin software (version 1.50; Jasco). Results are expressed as μM/mg of tissue.

#### Enzyme-Linked Immunosorbent Assays (ELISA) Quantifications

Cys-C levels were quantified in plasmatic samples by using Rat Cys-C (Enzyme-Linked Immunosorbent Assays) ELISA kit (Fine Test, Wuhan Fine Biotech Co., Ltd., Wuhan, Hubei, China), according to manufacturers’ instructions. In order to avoid intra-assay variations, both standards and samples were analyzed in duplicate.

### Statistical Analyses

Statistical analyses were performed by using Two-way ANOVA for repeated measures followed by Šídák’s multiple comparisons test or Unpaired Student’s t-test, as required. Welch’s correction was applied to Unpaired Student’s t-test when F test indicated unequal variances. The statistical software used was GraphPad 9.0 for Windows (GraphPad Software, San Diego, CA, United States). For all tests, *p*-value was set at 0.05. Results are expressed as means ± standard error of the mean (S.E.M.).

## Results

### Effects of Aβ Administration on Pain-Related Behavioural Tests

**FIGURE 1:**
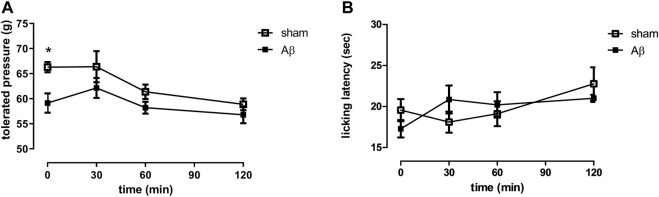
Effects of Aβ administration on paw pressure and cold plate tests. **(A)** Tolerated weight (g) in sham (n = 9) and Aβ-treated (n = 7) animals. Two-way ANOVA for repeated measures followed by Šídák’s multiple comparisons test, *p < 0.05 Aβ vs sham. **(B)** Licking latency (sec) in sham (n = 10) and Aβ-treated (n = 7) animals. Two-way ANOVA for repeated measures followed by Šídák’s multiple comparisons test, *p* > 0.05.

In order to evaluate the effects of Aβ administration in response to a noxious mechanical stimulus, we performed the paw pressure test (hyperalgesia-like measurement). Figure 1A showed that the pain threshold, expressed as grams of tolerated weight, was significantly decreased in Aβ-treated rats compared to sham rats (Figure 1A, Two-Way ANOVA for repeated measures, followed by Šídák’s multiple comparisons test, F _(3, 42)_ = 5.039, *p* < 0.05, time 0: Aβ vs sham). Moreover, we assessed the thermal pain sensitivity in Aβ-treated and sham rats by performing the cold plate test, based on a non-noxious cold stimulation (allodynia-like measurement). Our results showed no differences in cold hypersensitivity, expressed as latency to lick the paws, between the two experimental groups, although a slight trend to reduction could be appreciated (Figure 1B, Two-Way ANOVA for repeated measures, followed by Šídák’s multiple comparisons test, F _(3, 36)_ = 1.599, *p* > 0.05 n. s., Aβ vs sham).

### Effects of Aβ Administration on Social Memory

**FIGURE 2:**
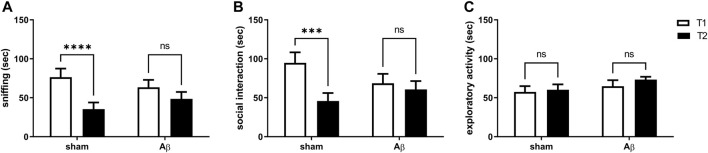
Effects of Aβ administration on social recognition paradigm. **(A)** Sniffing time (sec) in sham (n = 7) and Aβ-treated (n = 7) animals. Two-Way ANOVA for repeated measures followed by Šídák’s multiple comparisons test, ****p < 0.0001 Aβ vs sham. **(B)** Social interaction time (sec) in sham (n = 7) and Aβ-treated (n = 7) animals. Two-way ANOVA for repeated measures followed by Šídák’s multiple comparisons test, ***p < 0.001 Aβ vs sham. **(C)** Exploratory activity time (sec) in sham (n = 7) and Aβ-treated (n = 7) animals. Two-way ANOVA for repeated measures followed by Šídák’s multiple comparisons test, *p* > 0.05.

Here, we tested the effects of Aβ administration on social memory by carrying out the social recognition test. We found that sham rats reported a significant decrease in time spent sniffing and performing social interaction in the second exposition to a juvenile rats compared to first presentation, while no difference in both sniffing and social interaction between first and second encounter were retrieved in Aβ-treated rats (Figure 2A, Two-Way ANOVA for repeated measures followed by Šídák’s multiple comparisons test, F_(1,12)_ = 41.13, *p* < 0.0001 sham T2 vs sham T1, Figure 2B, Two-Way ANOVA for repeated measures followed by Šídák’s multiple comparisons test, F_(1,12)_ = 18.85, *p* < 0.001 sham T2 vs sham T1). In order to evaluate whether the results in time spent performing social exploration might depend from exploratory deficits, we scored total exploratory behaviours and we found no differences between trial 1 and trial 2 in both Aβ-treated and sham groups (Figure 2C, Two-Way ANOVA for repeated measures followed by Šídák’s multiple comparisons test, F_(1,12)_ = 1.109, n. s sham T2 vs sham T1).

### Effects of Aβ Administration on Hypothalamic Monoamines

**FIGURE 3:**
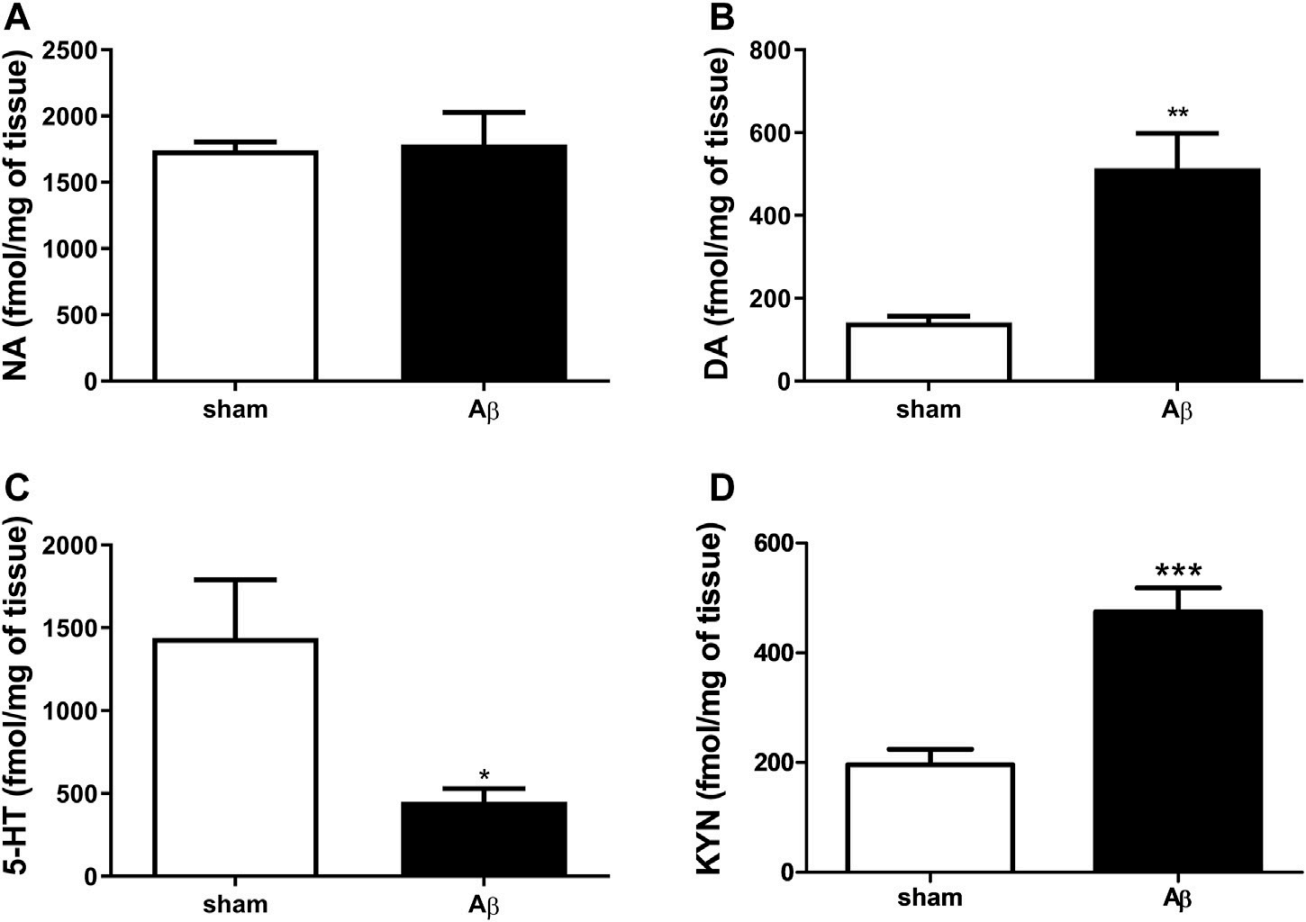
Effects of Aβ administration on hypothalamic NA, DA and 5-HT levels. **(A)** NA (fmol/mg of tissue) in sham (n = 5) and Aβ-treated (n = 6) animals. Unpaired Student’s t-test, *p* > 0.05. **(B)** DA (fmol/mg of tissue) in sham (n = 5) and Aβ-treated (n = 6) animals. Unpaired Student’s t-test, **p < 0.01 Aβ vs sham. **(C)** 5-HT (fmol/mg of tissue) in sham (n = 6) and Aβ-treated (n = 6) animals. Unpaired Student’s t-test, *p < 0.05 Aβ vs sham. **(D)** KYN (fmol/μl) in sham (n = 5) and Aβ-treated (n = 5) animals. Unpaired Student’s t-test, ***p < 0.001 Aβ vs sham.

In order to corroborate behavioural results with neurochemical analyses, we quantified NA, DA and 5-HT content in the hypothalamus. Our results show no difference in NA content between the two experimental groups (Figure 3A, Unpaired Student’s t-test with Welch’s correction, n. s., η^2^= 0.005560 Aβ vs sham), while DA was significantly increased in Aβ injected rats (Figure 3B, Unpaired Student’s t-test with Welch’s correction, *p* < 0.01 η^2^= 0.7808, Aβ vs sham). In addition, rats injected with Aβ showed a significant decrease in 5-HT levels (Figure 3C, Unpaired Student’s t-test with Welch’s correction, *p* < 0.05 η^2^= 0.5795, Aβ vs sham), while KYN levels were significantly increased (Figure 3D, Unpaired Student’s t-test, *p* < 0.001 η^2^ = 0.7456, Aβ vs sham) compared to controls.

### Effects of Aβ Administration on Excitatory-Inhibitory Neurotransmission in the Hypothalamus

**FIGURE 4:**
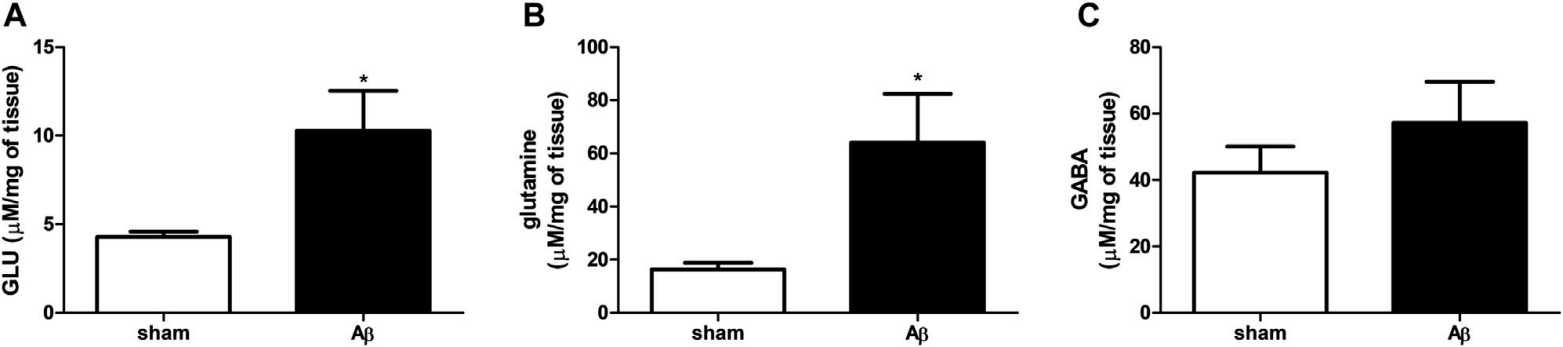
Effects of Aβ administration on hypothalamic GLU, glutamine and GABA levels. **(A)** GLU (μM/mg of tissue) in sham (n = 5) and Aβ-treated (n = 6) animals. Unpaired Student’s t-test, *p < 0.05. **(B)** Glutamine (μM/mg of tissue) in sham (n = 5) and Aβ-treated (n = 6) animals. Unpaired Student’s t-test, *p < 0.05. **(C)** GABA (μM/mg of tissue) in sham (n = 5) and Aβ-treated (n = 6) animals. Unpaired Student’s t-test, *p* > 0.05 Aβ vs sham.

We evaluated the effect of Aβ administration on hypothalamic GLU, glutamine and GABA levels. We found an increase in GLU and glutamine contents in Aβ-treated rats (Figures 4A,B, Unpaired Student’s t-test with Welch’s correction, η^2^ **=** 0.5718 and 0.5628, respectively, *p* < 0.05 Aβ vs sham). As regarding GABA quantifications, rats administrated with Aβ did not show any difference compared to sham rats (Figure 4C, Unpaired Student’s t-test with Welch’s correction, n. s. η^2^ **=** 0.1124, Aβ vs sham).

### Effects of Aβ Administration on Plasma 5-HT, 5-HT Turnover, KYN, MEL and Cys-C Levels

**FIGURE 5:**
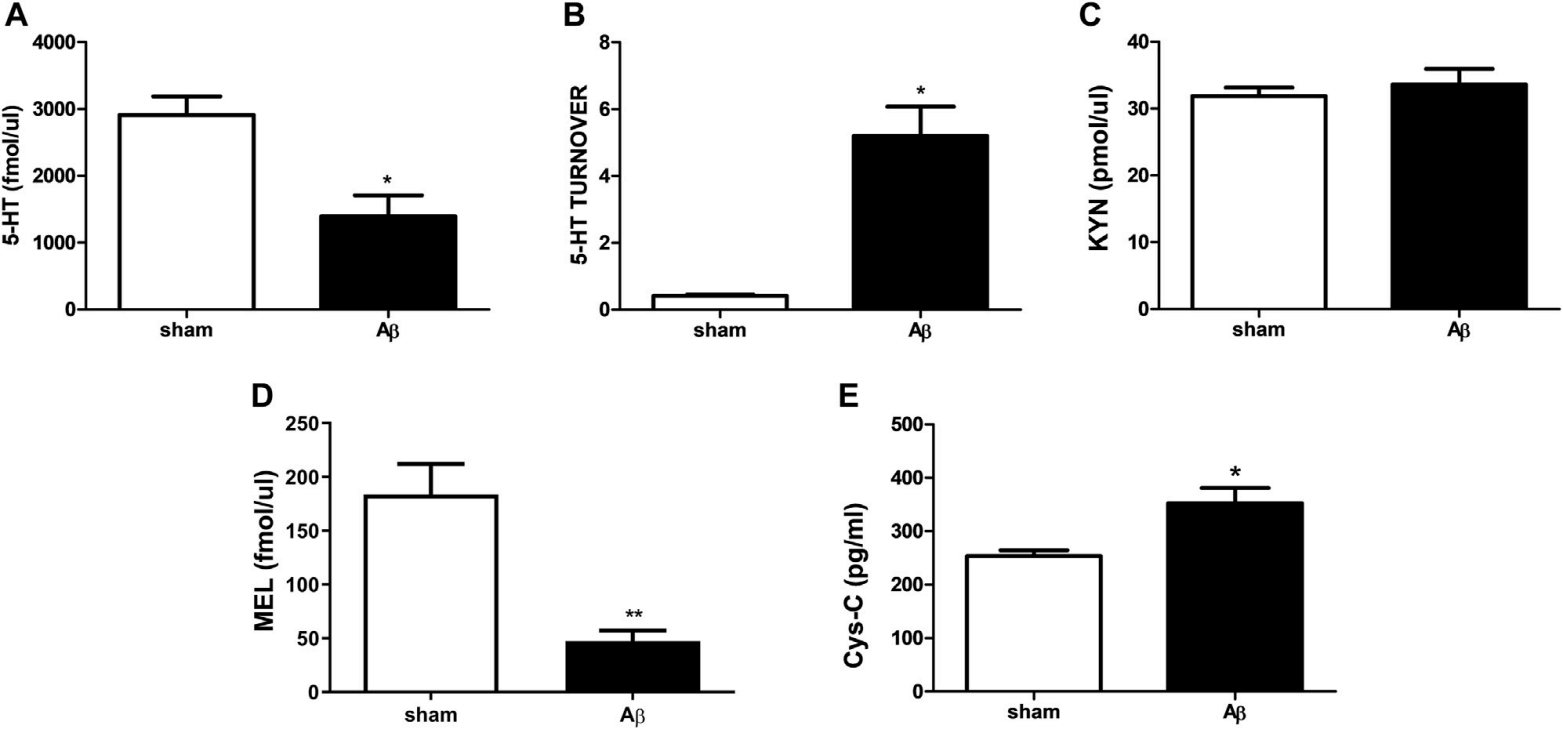
Effects of Aβ administration on plasma 5-HT, 5-HT turnover, KYN, MEL and Cys-C levels. **(A)** 5-HT (fmol/μl) in sham (n = 4) and Aβ-treated (n = 4) animals. Unpaired Student’s t-test, *p < 0.05 Aβ vs sham. (B) 5-HT turnover in sham (n = 4) and Aβ-treated (n = 4) animals. Unpaired Student’s t-test with Welch’s correction, *p < 0.05 Aβ vs sham. **(C)** KYN (pmol/μl) in sham (n = 5) and Aβ-treated (n = 5) animals. Unpaired Student’s t-test, *p* > 0.05 Aβ vs sham. **(D)** MEL (fmol/μl) in sham (n = 5) and Aβ-treated (n = 5) animals. Unpaired Student’s t-test, **p < 0.01 Aβ vs sham. **(E)** Cys-C (pg/ml) in sham (n = 5) and Aβ-treated (n = 5) animals. Unpaired Student’s t-test, *p < 0.05 Aβ vs sham.

In Figure 5, peripheral alterations after Aβ injection have been determined. In particular, we measured 5-HT and its turnover in plasma samples of Aβ-injected and sham rats, together with KYN, MEL and Cys-C content. Concerning 5-HT, rats treated with Aβ showed a significant decrease in plasmatic levels compared to sham (Figure 5A, Unpaired Student’s t-test with Welch’s correction, η^2^ = 0.6862, *p* < 0.05 Aβ vs sham). In addition, an increased 5-HT turnover was found in Aβ-treated rats compared to sham ones (Figure 5B, Unpaired Student’s t-test with Welch’s correction, η^2^ = 0.9085, *p* < 0.05 Aβ vs sham). No difference was found between groups in KYN levels (Figure 5C, Unpaired Student’s t-test, η^2^ = 0.03343, *p* > 0.05 n. s.), while MEL was significantly reduced in Aβ-treated rats (Figure 5D, Unpaired Student’s t-test**,** η^2^ = 0.7742, *p* < 0.01 Aβ vs sham). As regarding plasma Cys-C content, our results reported a significant increase in animals administrated with Aβ compared to sham rats (Figure 5E, Unpaired Student’s t-test, η^2^ = 0.5641, *p* < 0.05 Aβ vs sham).

### Effects of Aβ Administration on Urinary 5-HT, 5-HT Turnover, KYN, and MEL Levels

**FIGURE 6:**
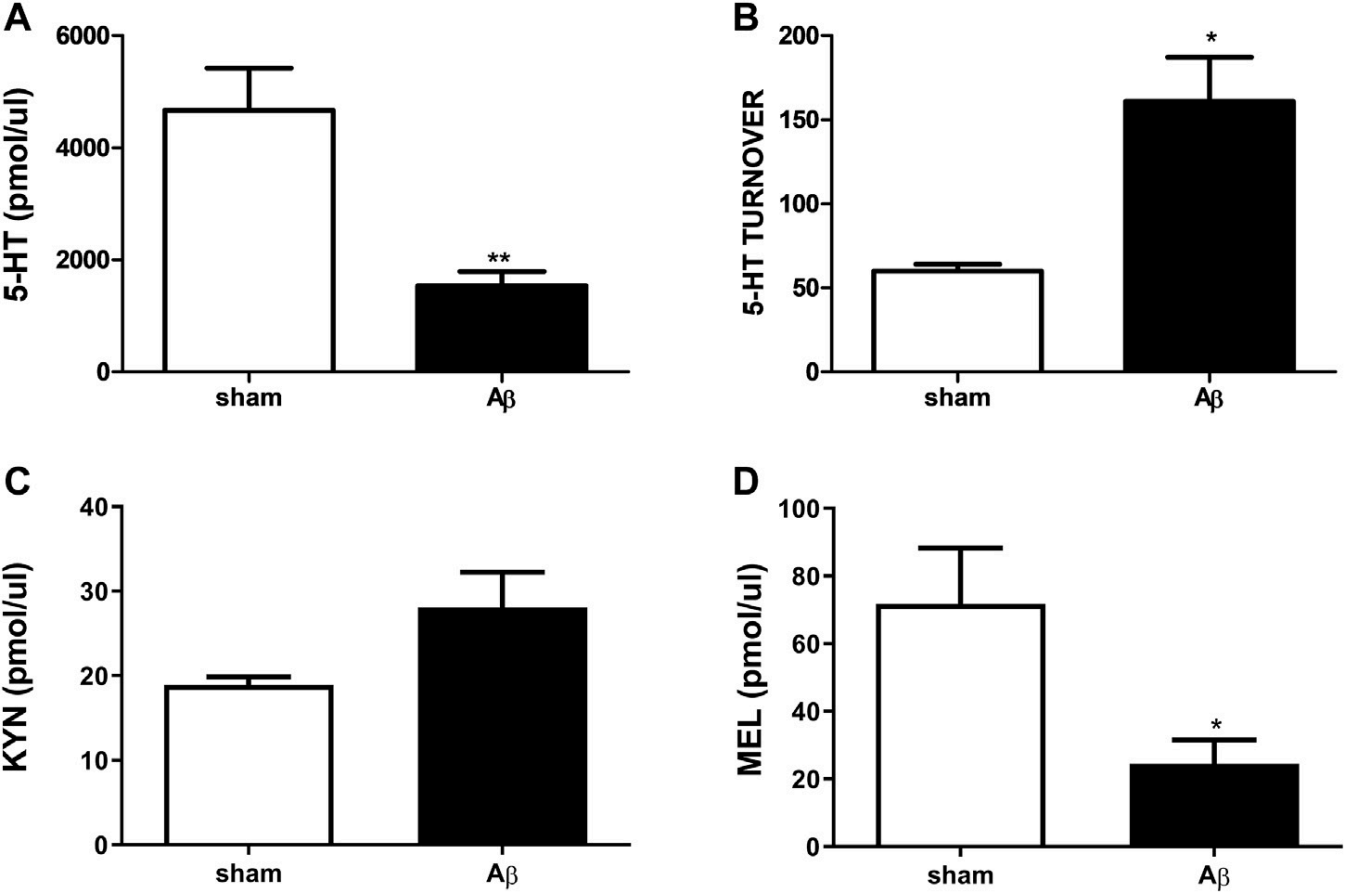
Effects of Aβ administration on urinary 5-HT, 5-HT turnover, KYN and MEL. **(A)** 5-HT (pmol/μl) in sham (n = 4) and Aβ-treated (n = 4) animals. Unpaired Student’s t-test, **p < 0.01 Aβ vs sham. **(B)** 5-HT turnover in sham (n = 4) and Aβ-treated (n = 4) animals. Unpaired Student’s t-test with Welch’s correction, *p < 0.05 Aβ vs sham. **(C)** KYN (pmol/μl) in sham (n = 5) and Aβ-treated (n = 5) animals. Unpaired Student’s t-test, *p* > 0.05 Aβ vs sham. **(D)** MEL (pmol/μl) in sham (n = 5) and Aβ-treated (n = 5) animals. Unpaired Student’s t-test, *p < 0.05 Aβ vs sham.

As shown in Figure 6, urinary concentrations of 5-HT and its turnover, KYN and MEL after Aβ or its vehicle injection were determined. Concerning 5-HT, rats treated with Aβ showed a significant decrease in its plasmatic levels compared to sham (Figure 6A, Unpaired Student’s t-test with Welch’s correction, η^2^ **=** 0.7247, *p* < 0.05 Aβ vs sham). In addition, an increased 5-HT turnover was found in Aβ-treated rats compared to control (Figure 6B, Unpaired Student’s t-test with Welch’s correction, η^2^ **=** 0.8237, *p* < 0.05 Aβ vs sham). No difference was found between groups in KYN levels (Figure 6C, Unpaired Student’s t-test, η^2^ = 0.5078, *p* > 0.05 n. s.), while MEL was significantly reduced in Aβ-treated rats (Figure 6D, Unpaired Student’s t-test, η^2^ = 0.4647, *p* < 0.05 Aβ vs sham).

## Discussion

In the present work, we aimed at evaluating if the rat model of Aβ-induced toxicity might represent a nonclinical model able to provide reliably probabilistic estimates of translation into human biology, according to the need of precision medicine.

Here, we demonstrated showed that in rats exposed to central administration of Aβ peptide, a rodent model we have previously shown validated useful for mimicking the symptomatology of early phase AD (Colaianna et al., 2010; Morgese et al., 2014), also showed impairment in social memory, along with reduced 5-HT content and enhanced GLU, DA and KYN at hypothalamic level. In addition, increased mechanonociception, but not thermal nociception, was evident after central Aβ administration.

This event might likely be due to increased inflammation caused by the peptide. Indeed, mechanonociception seems to be specifically dependent on phospholipase A2 (PLA2) activation, arachidonic acid release and production of pro-inflammatory cyclooxygenase (COX) derivatives (Meller, 1994). In keeping with this hypothesis, we have previously shown, in the same animal model, that the icv injection of Aβ peptide increased the expression of the inducible COX-2 enzyme (Mhillaj et al., 2018). Furthermore, it has been shown that expression of COX-2 as well as prostaglandin E2, can be enhanced after phosphorylation of MAPKs and NF-κB induced by oxidative stress (Onodera et al., 2015). In our experience, we have found that Aβ-induced toxicity is also occurring through the increased oxidative biomarker production and NF-κB expression (Morgese et al., 2021a; Morgese et al., 2021b), therefore we can speculate that alterations in oxidative and inflammatory status migh represent putative factors that predispose Aβ-treated rats to increased mechanical pain susceptibility.

Over the past few decades, a pivotal role for GLU in pain sensation and transmission has been increasingly proposed, and, in this regard, novel pain medications targeting this excitatory system are in the spotlight. Thus, the role played by supraspinal mGluRs to pain modulation, including the descending pain pathway that involves also the hypothalamus, is an emerging field of study (Pereira and Goudet, 2018). In this regard, it is well known that neural populations of the descending inhibitory pain pathway originate in the amygdala and in the hypothalamus and project to the spinal cord and other relevant areas (Rea et al., 2007). Glutamate and glutamatergic receptor activation in this area exerts a fine tune on the release of hypothalamic factors, including Substance P, crucial for pain chronicity (Zieglgansberger, 2019), and GABA. Thus, this model may be also suitable for the evaluation of novel drugs acting on these targets, future investigations are surely warranted.

Interestingly, hypothalamic dopaminergic tone was increased in Aβ-treated rats. Previous reports have evaluted hypothalamic-spinal dopaminergic system on pain modulation, however the role played by this neurotrasmitter in such a context has not been completely unravelled. Indeed, activation of DA receptors seems to differently interact with nociception and in particular, D2-like agonism has been linked to analgesia specific to mechanonociception, but not thermonociception (Almanza et al., 2015). It is worth to note that, in patients early diagnosed with AD, reduction of D2 receptors has been reported in brain areas associated with emotional control, such as the hippocampus, but also in brain areas deputed to control motor coordination such as the nigrostriatal pathways (Pan et al., 2019). Therefore, we can speculate that such an increase in DA can be related to reduced D2 receptors, leading to enhanced DA tone and higher D1 activation. On the other hand, Aβ has been shown to modulate the release of factors implicated in the response to stress, such as corticotropin releasing factor (CRF) (Lv et al., 2020). Elevated glucocorticoids can trigger dopamine release (Czyrak et al., 2003; Belujon and Grace, 2017), while GLU can induce CRF release (Herman et al., 2004). In addition, stress response is correlated with Aβ release and the peptide, in turn, controls HPA functioning (Morgese et al., 2017). In addition, along with the well defined decline of cognitive and emotional domains, also functions under hypothalamic control are dysfunctional in these patients, such as the disruption of circadian rhythms (Baloyannis et al., 2018). Indeed, reduced levels of MEL were identified at both plasma and urine in our model. This evaluation, in the light of individuating novel peripheral biomarkers useful for monitoring AD progression or efficacy of used AD medications might represents a valid advantage also for the purpose of precision medicine. In particular, MEL is a multifunctional hormone involved not only in the regulation of circadian rhythm, but also with recognized anti-oxidant and anti-amyloidogenic (He et al., 2010) properties, proposed as a possible factor for contrasting AD progression (Hossain et al., 2019). Indeed, MEL has been suggested as an early biomarker for detecting the first stages of AD (Wu et al., 2003; Hossain et al., 2019). Furthermore, MEL was shown to suppressed NF-κB, an ubiquitary located transcriptional effector of inflammatory mediators, whose activation lead to higher prostaglandin and nitric oxide levels contributing to the development of hyperalgesia (Petho and Reeh, 2012). In this regard, we have very recently shown, in mice, that the icv injection of Aβ led to increased expression of this factor (Morgese et al., 2021b). In addition, MEL can prevent IL-6 release NF-κB-induced in Aβ-treated brain slices (Lau et al., 2012) and memory loss secondary to NF-κB activation (Shen et al., 2007). Pro-inflammatory biomarkers, such as cytokines, are able of modulating neuronal activity by promoting the release of neuroactive molecules from glia or the endothelium, including GLU (Vezzani and Viviani, 2015). Furthermore, chronic inflammation is able to decrease central 5-HT tone (Couch et al., 2013) also in condition of pseudoinflammation, an inflammatory state in absence of pathogens, associated with enhanced Aβ production and depressive-like behavior in rats (Morgese et al., 2020). Lower cerebral 5-HT levels leads to alteration in functioning of descending pain pathways, indicating that impairment in mood and emotions mediated by brainstem areas are important in determining the levels of pain. In particular, the emotional and autonomic aspects of nociceptive processing crucially implicated the parabrachial area. This important supraspinal target relays to the hypothalamus and amygdala, thus driving the descending modulatory pathways through the rostroventral medulla, resulting in a spino–bulbo–spinal loop. Indeed, the effectiveness of pain relievers depends on 5-HT levels and represents a mechanism whereby emotions can alter pain perception (Suzuki et al., 2004). As confirmation, pharmacological modulation that correspond to higher 5-HT tone has been shown to be useful for reverting pain-like behaviors in pertinent animal models (Bobinski et al., 2015). Indeed, in our model, we found that 5-HT was reduced in the hypothalamus of treated rats. Therefore, such different outcomes, retrieved in the behavioral paradigms used, might depend on the selective activation of glutamatergic and serotonergic pathways associated with inflammatory state. In this regard, it has been reported that inflammation (Tu et al., 2005), as well as increased corticosterone levels (Luo et al., 2017), can lead to enhaced metabolism of tryptophan toward KYN production at hypothalamic level. Moreover, it has been reported that KYN metabolites can directly act on GLU receptors, thus influencing excitatory neurotransmission and stimulating an increase in GLU release (Schwarcz, 2016). In addition, plasma and urinary content of 5-HT and 5-HT turnover were reduced in our model. This kind of alterations have been reported also in depressed patients (Mitani et al., 2006) and might represent a possible non invasive biomarkers for monitoring drug efficacy.

In keeping with this hypothesis, Aβ administration was able to alter other relevant biological peripheral parameters, such as Cys-C (Zhai et al., 2016), involved in both depression and AD (Sundelof et al., 2008; Minev et al., 2010). This molecule is a cysteine protease inhibitor, expressed either centrally (Lofberg and Grubb, 1979) or peripherally. This enzyme has been proposed as a biomarker of renal insufficiency and it has been shown to correlate with depressive symptoms in the elderly with normal renal function (Minev et al., 2010). Accordingly, a greater Cys-C levels were associated with an increased risk of new diagnosis of depression in subjects with normal renal function (Li H. et al., 2019). Furthermore, cognitive decline and AD are associated with renal impairment in human (Zhang et al., 2020) and in animal model of AD (Nakagawa et al., 2017). In further support of this evidence, it has been shown that Cys-C and Aβ peptide co-localize in brains of AD patients (Sastre et al., 2004) and polymorphisms of the Cys-C gene are associated with an increased risk for AD (Finckh et al., 2000; Chuo et al., 2007). Furthermore Cys-C levels directly correlate with cognitive impairment in the elderly (Yaffe et al., 2008). Our data are in line with these clinical evidences, considering that higher systemic Cys-C levels were found here and impairment of memory were previuosly shown in our model (Mhillaj et al., 2018). Cys-C was reported to be higher also in chronic pain patients and persistent pain can induce the synthesis and the release of Cys-C in dorsal spinal cord able to outflow to the brain (Mannes et al., 2003).

In conclusion, we found that in our model of depressive-like phenotype, induced by central administration of Aβ peptide, a state of enhanced pain perception was present. This increased susceptibility to mechanical pain was accompanied by impaired central and peripheral neurotransmission. In turn, also alterations in peripheral biomarkers, both linked to depression and AD, were evidenced. These outcomes also support the hypothesis that this animal model can be a suitable tool for investigating comorbid conditions associated with the early phase AD.

After a long search for new drugs for this condition, AD is still a fatal global epidemic. However, research in recent decades has focused its attention on the development of drugs capable of counteracting this complex pathology without seeking specific treatments for specific symptoms. This animal model could therefore result in a useful tool displaying some AD endophenotypes on which a targeted pharmacological strategy could be pursued in a tailored manner.

## Data Availability

The raw data supporting the conclusion of this article will be made available by the authors, without undue reservation.
